# Expanding the horizons of facial mimicry in chimpanzees (*Pan troglodytes*): new insights into diverse signal types and contexts

**DOI:** 10.1093/cz/zoaf077

**Published:** 2025-12-26

**Authors:** Brittany N Florkiewicz, Teddy Lazebnik

**Affiliations:** Department of Psychology, Lyon College, 2300 Highland Road, Batesville, AR, 72501, USA; Research Center for Human-Animal Interaction, College of Veterinary Medicine, University of Missouri, Clydesdale Hall, 900 E Campus Dr, Columbia, MO 65201, USA; Department of Information Systems, University of Haifa, Haifa 3498825, Israel; Department of Computing, Jönköping University, Gjuterigatan 5, 553 18 Jönköping, Sweden

**Keywords:** chimpanzees, communication, facial mimicry, facial signals, rapid facial mimicry

## Abstract

Facial mimicry (FM) is an evolved mechanism in social mammals that potentially fosters emotional and physical connections through the production of congruent facial signals. Most research on FM has primarily examined relaxed open-mouth facial displays in the context of play. However, if the primary function of FM is to enhance the accuracy of predicting others’ behaviors, it stands to reason that FM would extend to various contexts and signal types. The aim of our current study was to determine whether rapid FM also occurred in different contexts and types of facial signals in a captive troop of chimpanzees. We hypothesized that rapid FM would be more prevalent in social contexts where evidence of FM has been reported across multiple species (hypothesis 1), as well as in the facial signal types associated with these contexts (hypothesis 2). Additionally, we expected that rapid FM would occur more frequently with simpler facial signals composed of fewer distinct muscle movements (hypothesis 3). We tested our three hypotheses across ten different contexts and with nine types of facial signals. We employed new computational methods that measure the number and proportion of facial muscle movements mimicked, which we refer to as RMsize and RMratio, respectively. These methods help us differentiate between types of rapid FM (exact matching versus type matching). We found no evidence to support our three hypotheses, suggesting that FM may be a more widespread mechanism than previously believed.

## Graphical Abstract

**Graphical Abstract:**
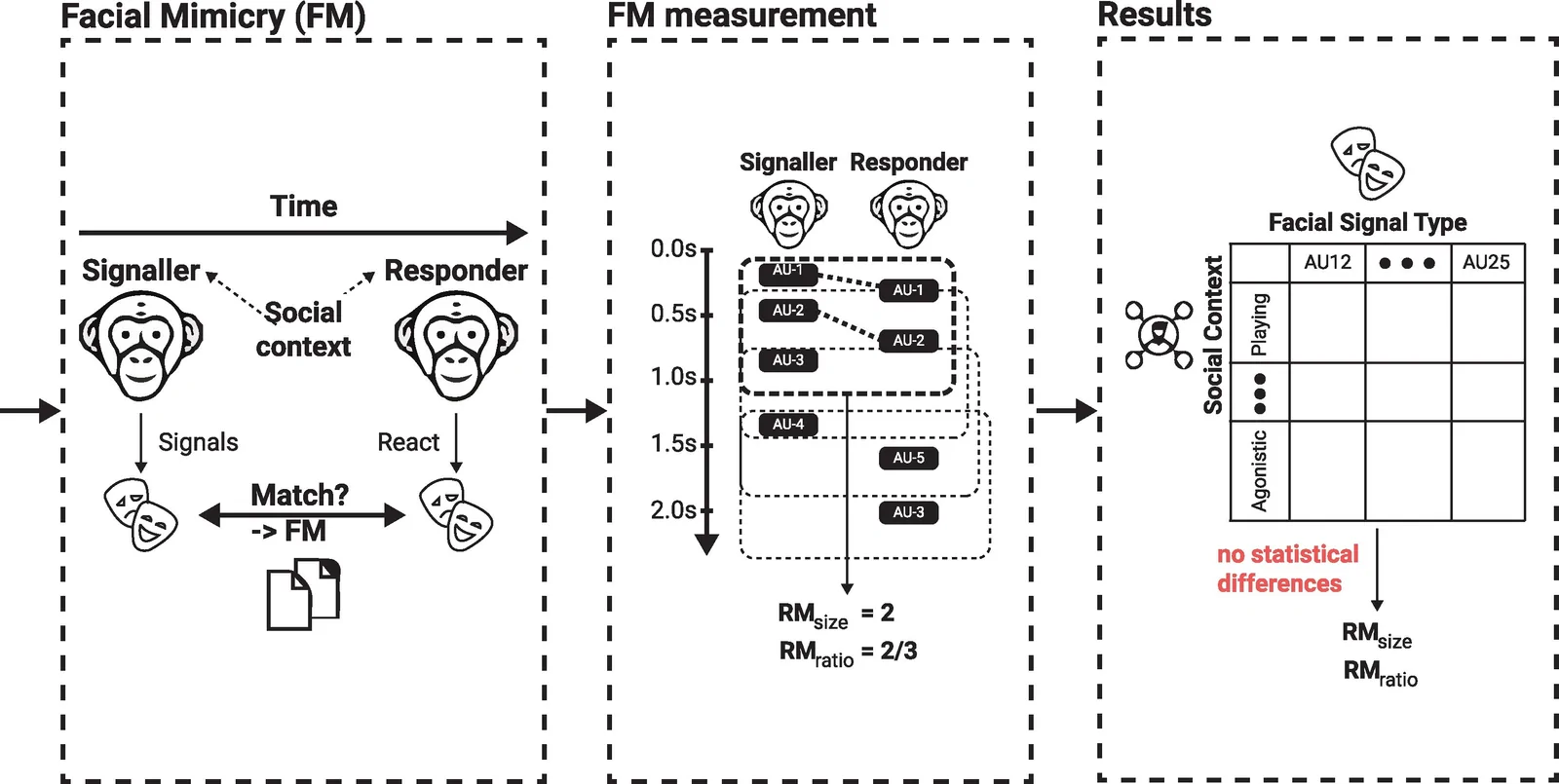

Facial mimicry (or FM) is a widespread mechanism that has evolved to enable social mammals (like primates) to connect both physically and potentially emotionally with conspecifics (Davila-Ross and Palagi 2022). FM is a quick and automatic process where an individual produces a similar facial signal after seeing the same signal being produced by another individual (Mendl and Paul 2020; Facondini et al. 2024). It is believed that FM results from perception-action mechanisms (PAMs) which allow individuals to automatically align observed actions with their own motor systems (Moody et al. 2007). The proposed function of FM and evidence for PAMs is based on the presence of a mirror neuron system (MNS) in multiple species. In nonhuman primates (such as macaques, *Macaca mulatta*), the F5 region of the premotor cortex and a portion of the inferior parietal lobule activate not only during the execution of an action but also when observing the same action performed by others (Gallese et al. 1996; Caggiano et al. 2011). In humans, the MNS includes more than a dozen specific brain regions, including those involved in emotion processing (Likowski et al. 2012). Additional areas may be activated depending on the type of facial signal observed or produced, such as the activation of the hippocampus when viewing and producing angry faces (Likowski et al. 2012). The MNS provides a possible neural framework for FM among primates, as it allows individuals to perceive and mimic similar facial muscle movements (Tramacere and Ferrari 2016).

Some researchers suggest that FM and PAMs are essential in regulating one's own emotional experiences and behaviors, a concept known as the facial feedback hypothesis (Andreasson and Dimberg 2023). However, the evidence linking facial muscle movement and emotional experience has been questioned (Buck 1980; McIntosh 1996). Previous studies have suggested a potential connection between socio-emotional experiences and facial muscle movements (Estow et al. 2007; Moody et al. 2007). Research indicates that modulating facial muscle movements associated with specific emotions can alter emotional intensity among humans (Laird 1974; McIntosh 1996). In nonhuman primates, the intensity of emotions, such as pain, is often reflected in the intensity of their facial muscle movements (Mota-Rojas et al. 2025). However, the connection between emotional experiences and facial muscle movements has been challenged in both humans and nonhuman primates. This skepticism arises because facial muscle movements can occur without emotional arousal and, conversely, emotional arousal can happen without corresponding facial movements (McIntosh 1996). Facial muscle movements in primates may be inhibited or controlled, partially supported by studies showing audience effects with facial signals (Fridlund 1991; Crivelli and Fridlund 2018; Kret et al. 2020). Additionally, socio-cultural factors have been shown to influence the connection between facial signals (and their associated muscle movements) and emotional arousal in humans (Cordaro et al. 2018), indicating a more intricate relationship between movement and emotion. The link between FM and emotional experience has also been questioned, as FM may occur independently of any emotional experience (McIntosh 1996).

At the very least, it appears that FM may play an important role in regulating behavior among mammals like primates, as facial signals and their corresponding muscle movements help individuals predict and respond to the behaviors of others (Fridlund 2002; Waller et al. 2017). It has been suggested that FM evolved primarily to reduce prediction errors related to others' behavior (through the matching of internalized and externalized behavioral states), resulting in improved regulation of social interactions (Kret and Akyüz 2022). Indeed, FM appears to be linked to behavioral coordination in social interactions among primates (Mancini et al. 2013; Palagi et al. 2019a, 2019b). To date, FM has been identified in multiple species of primates and carnivores (for a review, see Davila-Ross and Palagi 2022; Cordoni et al. 2024; Maglieri et al. 2024). These studies have revealed that multiple kinds of FM exist based on two key measurements: (1) the speed of mimicking facial muscle movements, and (2) the quantity of individual facial muscle movements that are replicated (Fig. 1; see Palagi et al. 2019a, 2019b; Davila-Ross and Palagi 2022; Martvel et al. 2024). For example, the facial muscle movements associated with a specific facial signal can be mimicked quickly (within 1 s of viewing) or after a delay (up to 5 s after viewing; (Palagi et al. 2019a, 2019b; Davila-Ross and Palagi 2022; Martvel et al. 2024). These forms of FM are often referred to as ***rapid FM*** and ***delayed FM***, respectively (Mancini et al. 2013; Palagi et al. 2019a, 2019b). Rapid FM is used to convey and coordinate immediate behaviors, while delayed FM is employed to manage later aspects of social interactions (Palagi et al. 2019a, 2019b). Primates can either mimic all of the facial muscle movements produced after viewing a given facial signal (referred to as ***exact matching FM***) or mimic only certain movements that are essential for generating a stereotyped signal (referred to as type matching FM; Taylor et al. 2019; Bresciani et al. 2022; Martvel et al. 2024). As the number of mimicked facial muscle movements increases after the production of the original signal (within 1 s for rapid facial mimicry [RFM] and within 5 s for delayed facial mimicry [DFM]) the likelihood of extending the social interaction also increases (Mancini et al. 2013; Bresciani et al. 2022; Cordoni et al. 2024).

**Figure 1:**
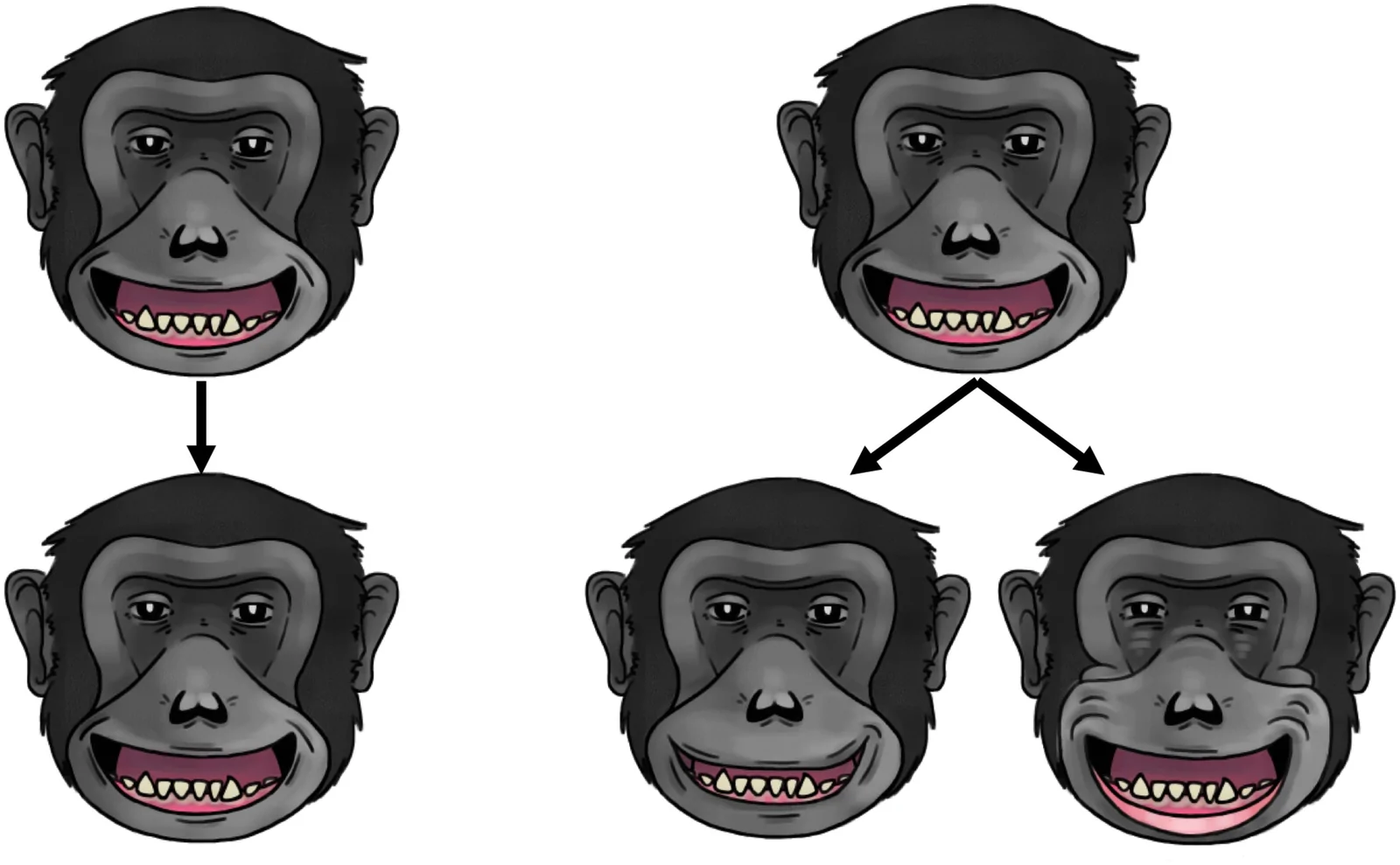
A visual example illustrating two types of facial mimicry (FM) using a relaxed open-mouth display: exact matching (left image set) and type matching (right image set). In exact matching, the respondent (bottom row) mimicks all the facial muscle movements of the signaler (top row). In contrast, type matching involves producing the same kind of facial signal (a relaxed open-mouth face) but with varying movements. Illustrations were created by the first author (B.N.F.).

Most studies on the different types of FM have focused primarily on relaxed open-mouth displays, characterized by lip parting and a relaxed jaw (Davila Ross et al. 2008; Mancini et al. 2013; Scopa and Palagi 2016; Palagi et al. 2019a, 2019b; Maglieri et al. 2020; Bresciani et al. 2022; Gallo et al. 2022; Cordoni et al. 2024; Facondini et al. 2024). However, one recent study involving bonobos (*Pan paniscus*) found evidence of FM during the production of bared-teeth displays (Palagi et al. 2020), which are produced by pulling the corners of lips back to reveal both rows of teeth (Van Hooff 1967; Maestripieri and Wallen 1997; Waller and Dunbar 2005; Clark et al. 2020; Kim et al. 2022). Both the relaxed open-mouth display and the bared-teeth display differ in their physical forms and contextual use. In chimpanzees (*Pan troglodytes*), relaxed open-mouth displays are often seen in play contexts, accompanied by behaviors such as rolling, tumbling, and feign biting (Waller and Dunbar 2005). In contrast, the bared-teeth display is seldom produced with these behaviors among chimpanzees (Waller and Dunbar 2005). In bonobos (*P. paniscus*), bared-teeth displays are commonly produced during sexual encounters, accompanied by behaviors such as genital rubbing and mounting (Palagi et al. 2020). If FM helps reduce prediction errors and improve social interactions, it stands to reason that FM would be more likely to occur in social contexts where misunderstandings could lead to physical conflict (as is sometimes the case in rough-and-tumble play; Palagi et al. 2016) or fewer reproductive opportunities (which, in turn, impacts direct fitness; Palagi et al. 2020).

FM may not be limited to relaxed open-mouth displays, bared-teeth displays, or “affinitive’ contexts (which includes playful and sexual encounters; Waller and Dunbar 2005) in nonhuman primates. For example, although evidence for FM has been noted in laughter and smiles in humans (considered homologous to the relaxed open-mouth display and bared teeth displays of nonhuman primates; Kim et al. 2022), there is also evidence for FM in facial displays of fear and anger during nonfriendly interactions (Dimberg 1982; Moody et al. 2007). These negative displays alert individuals to potential threats, encourage problem-solving, and facilitate social bonding (Nesse 1990). However, to our knowledge, FM has seldom been studied during the production of aggressive and fearful displays (such as the scream face; Parr et al. 2007, 2008) in nonhuman primates. It is also unclear whether FM can occur in “nonaffinitive’ social contexts, such as during fights or other situations that might lead to fighting (such as eating, especially in species with higher levels of documented feeding competition). It is important to study the prevalence of FM across different signal types and contexts simultaneously, as one facial signal type can be associated with multiple social contexts. For example, in nonhuman primates, the relaxed open-mouth display is primarily linked to playful situations (Davila Ross et al. 2008; Mancini et al. 2013; Scopa and Palagi 2016; Palagi et al. 2019a, 2019b; Maglieri et al. 2020; Bresciani et al. 2022; Gallo et al. 2022; Cordoni et al. 2024; Facondini et al. 2024). However, the relaxed open-mouth display can also be observed in neutral contexts, during grooming sessions, and as a submissive signal following aggressive encounters to encourage social bonding (Zhang et al. 2018; Murray et al. 2023).

The goal of our current study is to evaluate whether various types of facial signals and their associated muscle movements, beyond the relaxed open-mouth display and bared-teeth display, are subject to FM. In addition, we seek to determine if variations in FM are context-dependent. We focus our study specifically on chimpanzees because they have been found to produce a large and diverse repertoire of facial signals (Parr et al. 2005; Florkiewicz et al. 2024; Florkiewicz and Lazebnik 2025; Mahmoud et al. 2025) and demonstrate robust evidence of FM (Anderson et al. 2004; Campbell et al. 2009; Pressi et al. 2017; Palagi et al. 2019a, 2019b). Studying the use of FM in chimpanzees also provides deeper insights into how FM may differ among species with varying socio-ecologies. Given that FM has been widely observed in various species during play, as well as during sexual and grooming interactions, we anticipate that FM will be most prevalent in these contexts (***hypothesis 1***) and among facial signal types that typically accompany interactions in these social contexts (***hypothesis 2***). In a previous study, it has been found that chimpanzees possess larger and more complex facial signaling repertoires compared to other apes, such as hylobatids (Florkiewicz et al. 2024). This difference may arise from chimpanzees' use of highly flexible facial gestures in their communication, allowing them to navigate various kinds of interactions with conspecifics (Florkiewicz and Campbell 2021). Thus, hylobatids, in contrast, might depend more on other socio-communicative mechanisms (such as FM) which can be effectively communicated through simpler facial signals made up of fewer discrete muscle movements and a smaller overall communicative repertoire (Florkiewicz et al. 2024). The idea is that as the number of facial muscle movements increase, the probability of error also increases. Given these conclusions, we predict that FM will have an inverse correlation with the number of facial muscle movements within a given facial signal (***hypothesis 3***). In our current study, we focused on identifying instances of ***RFM)*** across signal types and social contexts in chimpanzees. RFM occurs when one chimpanzee mimics the facial muscle movements of another chimpanzee's facial signal within 1 s of viewing. In contrast to other forms of FM (such as DFM), RFM is the most widely studied, enabling us to draw comparisons with previously published studies.

## Materials and methods

### The dataset

In our current study, we utilize a dataset on chimpanzee facial signals that we previously published, initially aimed at exploring the combinatorics and complexity of these signals. We recognize that there are several ethical issues in the scientific community regarding the recycling of datasets, including practices such as “salami slicing.” However, in our current study, we employ new computational techniques with a large and diverse facial signaling dataset to explore novel research questions that we have not previously investigated. We aim to be transparent about the process by citing previous articles and clearly identifying which variables were already operationalized and which ones are new.

The previously published dataset is available as an electronic supplement in our open-access article (Florkiewicz and Lazebnik 2025), and is also available in our electronic supplement. Since these data were obtained through noninvasive behavioral observations, and we did not make use of any new animal subjects, evaluation by our affiliated Institutional Animal Care and Use Committees (IACUC) was waived. Data were collected using a combination of focal and opportunistic sampling methods with a captive troop of 18 chimpanzees housed at the Los Angeles Zoo from 2017 to 2019. Additional information about these chimpanzees can be found in the electronic supplement. Video footage was coded for the following variables, which we also use in our current study:

1. **Communicative interaction:** Defined as a social interaction involving one (or more) facial signals being exchanged between two (or more) chimpanzees. Video clips can feature two or more communicative interactions at a given time. We retained this variable in our present study to ensure the accurate identification of instances of RFM that occur within a social interaction between chimpanzees. Since some communicative interactions overlap or occur sequentially, the risk of misidentifying cases of RFM between communicative interactions is high. It is important to note that this dataset includes only communicative interactions where the start, duration, and end of the entire interaction were captured on camera, with all individuals involved clearly visible. Interactions that were recorded too late, cut off too soon, or where the individuals were not clearly in view were excluded from the dataset. Using these criteria, we identified a total of 685 communicative interactions.
  - ***Generalized behavioral context:*** For each communicative interaction, one of ten behavioral contexts that best encompassed the behaviors and facial signals produced during the interaction was assigned. The following ten behavioral contexts were considered: affiliation, agonism, arousal, feeding, grooming, locomotion, playing, resting, sex, and unsure/unknown (Pollick and de Waal 2007; Florkiewicz and Campbell 2021). We retained this variable to test hypothesis 1. An ethogram detailing these contexts and their associated behaviors is available in the electronic supplement. Information about the number of observations per context can also be found in the electronic supplement.
2. **Facial signal:** Defined as one (or more) facial muscle movements produced by a signaler during communicative interactions. Muscle movements associated with biological maintenance activities, such as chewing, were excluded. The onset (when facial muscle movements are first produced by the signaler), duration, and offset of each facial signal (when the movements cease) in every video were recorded. We retained this variable to assess instances of facial signaling and to assist with identifying RFM. In the original dataset, 1,090 facial signals were identified across the 685 communicative interactions.
  - ***Chimpanzee ID:*** The identity of each chimpanzee that produced a facial signal was recorded. We retained this variable to ensure accurate identification of cases of RFM in situations where one chimpanzee produces a facial signal shortly after the previous one. RFM should only occur between two chimpanzees (the signaler and responder).
  - ***Signaling reason:*** Information on whether the facial signal was produced by the chimpanzee who initiated the communicative interaction (signaling) or by another chimpanzee in response to viewing a facial signal (responding) was recorded. We retained this variable to ensure accurate identification of cases of RFM. As previously mentioned, RFM should only occur between a signaler and recipient/responder.
  - ***Facial signal type:*** All facial signals were categorized into commonly referenced behavioral typologies based on similarities in key facial muscle movements. An ethogram detailing these signal types and their associated facial muscle movements is available in the electronic supplement. The following nine facial signal types were considered: ambiguous face, bared-teeth face, lipsmacking face, lower lip relaxer face, pant hoot face, relaxed open-mouth face, pout face, raspberry face, scream face (Parr et al. 2007, 2008; Florkiewicz and Campbell 2021). We retained this variable to test hypothesis 2. Information about the number of observations per facial signal type can be found in the electronic supplement.
  - ***Action unit (AU) combination:*** Each facial signal was coded according to the guidelines of ChimpFACS (Parr et al. 2007; Vick et al. 2007), where all facial muscle movements (referred to as AUs) are identified and assigned a unique AU code. The combination of AUs generates a facial signal, with all AUs coded at the peak of their production. We retained this variable because it is essential for identifying instances of RFM. By using ChimpFACS coding protocols, we can evaluate when specific facial muscle movements are mimicked within the 1-s timeframe. Additionally, we can analyze how many of the movements in the signal are mimicked to differentiate exact versus type FM. Finally, using ChimpFACS helps categorize facial signals into typologies, distinguishing relaxed open mouth displays from other signals. A list of all AUs that were considered and coded can be found in the electronic supplement.
  - ***Action unit (AU) count:*** The number of unique AUs per facial signal was calculated. In our original study, this metric was used to evaluate the complexity of facial signals (Florkiewicz et al. 2024). We retained this variable to test hypothesis 3.

In our previous study, interobserver reliability was calculated using 10% of video clips obtained from 2018, which included 149 of 1,090 facial signals. Agreement was assessed for generalized behavioral contexts, as well as facial signal types and AU combinations informed by ChimpFACS coding. The primary coder of the original study (and first author of our current study, B.N.F.) worked with two additional researchers (M.W.C. and S.Y.) to assess agreement. B.N.F. and S.Y. are both certified to use ChimpFACS, and assessed agreement for facial signal types and AU combinations. B.N.F. and M.W.C. assessed agreement for generalized behavioral contexts. Agreement was assessed using percentages and Cohen's kappa (k) for facial signal types and generalized behavioral contexts. In prior studies on chimpanzees (Hobaiter and Byrne 2011; Florkiewicz and Campbell 2021), good agreement was indicated by percentages above 70.00% and kappa scores of 0.61 or higher. In this dataset, the percentage of agreement for facial signal types was 74.83%, while the percentage for generalized behavioral contexts was 74.00%. Cohen's Kappa scores were 0.679 for facial signal types and 0.661 for generalized behavioral contexts. For our AU combination coding, we evaluated agreement using Wexler's ratio, which is a standard practice in other studies using ChimpFACS (Parr et al. 2007). We calculated Wexler's ratio for each of our 149 facial signaling observations (and associated AU combinations) and used the average ratio to assess overall agreement. We established a minimum Wexler's ratio of 0.70 for what is considered “good” agreement, as this threshold is also the standard for passing FACS certification tests for both humans and nonhumans (Lewinski et al. 2014). Our average Wexler's ratio was 0.75, indicating good agreement between B.N.F. and S.Y.

### Rapid facial mimicry (RFM) analysis

Given the dynamic nature of facial signals and their variation throughout communicative interactions, expressions of RFM can fluctuate in terms of specific AUs and their timing. For example, imagine a chimpanzee (A) produces a facial signal that lasts for 5 s, consisting of AU12, AU25, and AU26. It is plausible that AU12 is the first facial muscle movement, occurring within the first second of the signal. Meanwhile, AU25 and AU26 might be produced together at the third second of the signal. This means that the 1-s window for identifying instances of RFM will vary based on when each individual facial muscle movement occurs. To mitigate these challenges, one can consider the temporal information associated with a set of facial signaling interactions, modeling it as a sequence. Under such formalization, analyzing the duration between the peak of each facial signal and the sequential order of AUs presents an instance of an auto-regression analysis. To identify instances of RFM, we represent ChimpFACS-coded data as a sequence of events. Each event, *e*, is defined as a tuple *(c, a, t)*, where *c* denotes the participant (signaler *S* or responder *R*), *a* represents a specific facial signal, and *t* indicates the timestamp in seconds relative to the start of the video. In accordance with established RFM literature (Palagi et al. 2015), we constrained our analysis to a temporal window of *τ* = 1 second(s) to capture only potential RFM events.

To account for variation in the extent to which a chimpanzee mimics the AUs of a conspecific's facial signal (i.e., differences in type versus exact RFM), we adopted the computational approach proposed in (Martvel et al. 2024). This involved quantifying the number (RMsize) and proportion (RMratio) of AUs that were mimicked within a *τ* seconds window (*w*) between the responder (*R*) and signaler (*S*):


$$RMsize(w):=|S(w)|\cap |R(w)|,\;RMratio:=\frac{\left|S(w)|\cap |R(w)\right|}{\left|S(w)|\cup |R(w)\right|}.$$

Revisiting the previous example involving AU12, AU25, and AU26: if a responding chimpanzee (B) produces AU25 and AU26 by the fourth second of the original signaler (A)'s signal production (i.e., within 1 s of viewing AU25 + AU26), but does not produce AU12 at all, then we would calculate an RMsize of 2 and an RMratio of 0.667. While not commonly utilized in FM research, these two measures can be beneficial for identifying the likelihood of FM in datasets not specifically designed for this study.

It is important to note that all facial signals and their associated Action Units (AUs) exchanged between both signalers and recipients during a communicative interaction are evaluated for RMsize and RMratio measures, provided that a facial signal was produced in response to another facial signal. This means that in a given communicative interaction, an RMsize and RMratio value of 0 is possible if no instances of RFM occurred. Instances in which only one facial signal was produced during a communicative interaction, or where only the signaler produced facial signals without any response from the recipient, were excluded from our analysis, as RFM should only occur in response to viewing a facial signal. We only examined facial signals directed at another chimpanzee, not those produced in isolation or toward the troop as a whole. It is also important to recognize that congruent facial muscle movements may occur even when the types of facial signals are significantly different (i.e., congruency is not related to type or exact FM). This could be particularly evident in specific facial muscle movements, such as AU25 (lips part) and AU26 (jaw drop), which are commonly present in all “mouth-opening” facial signals. However, chimpanzee facial signals typically involve a variety of distinct facial muscle movements (Florkiewicz et al. 2024). With thousands of potential facial muscle movement combinations (Mahmoud et al. 2025) that can vary in timing, and considering the limited time frame of our observations using RMsize and RMratio, the likelihood that all our findings of congruent facial muscle movements arise purely by chance is very low.

### Comparisons

Consistent with previous studies (Mancini et al. 2013), we assessed differences in average RMsize and RMratio scores for generalized behavioral contexts and facial signal types using Mann–Whitney *U* tests in base R (Chambers 2008). Specifically, we group and compare contexts associated with previous documented evidence of RFM such as play, sex, grooming, and affiliation (Davila Ross et al. 2008; Mancini et al. 2013; Scopa and Palagi 2016; Zhang et al., 2018; Palagi et al. 2019a, 2019b; Maglieri et al. 2020; Palagi et al. 2020; Bresciani et al. 2022; Gallo et al. 2022; Cordoni et al. 2024; Facondini et al. 2024; Martvel et al. 2024) with other contexts not previously described (e.g., agonism, arousal, feeding, locomotion, resting, and situations where the context is unsure/unknown). Additionally, we group and compare facial signal types that are known to be subject to RFM, including relaxed open-mouth faces and bared-teeth faces (Palagi et al. 2019a, 2019b; Palagi et al. 2020), in relation to other facial signal types, such as ambiguous faces, lip-smacking faces, lower lip relaxed faces, pant-hooting faces, pout faces, raspberry faces, and scream faces. Finally, we qualitatively examine the categories of the number of AUs within a given facial signal, ranging from 1 to 9 or more. Our Python and R code, along with the raw data used to calculate RMsize and RMratio, can be found in the electronic supplement.

## Results

We calculated the average RMsize and RMratio scores based on the generalized behavioral context (Table 1), facial signal type (Table 2), and the number of AUs within a specific facial signal (Table 3). Generalized behavioral contexts previously examined (play, sex, grooming, and affiliation) exhibited no significant differences in their average RMsize and RMratio scores compared to other types of generalized behavioral contexts (Mann–Whitney *U* = 8, *P* = 0.454). Furthermore, facial signal types previously examined (relaxed open-mouth faces & bared-teeth faces) exhibited no significant differences in their average RMsize (Mann–Whitney *U* = 2, *P* = 0.186) and RMratio (Mann–Whitney *U* = 1, *P* = 0.111) scores compared to other types of facial signals. Upon examining the number of AUs in facial signals, there were no notable increases in RMsize or RMratio as the number of AUs decreased. In fact, as the number of AUs within a given signal increased, so did the value for RMsize (Table 3).

**Table 1:** Average RMsize and RMratio scores based on generalized behavioral context.

Generalized behavioral context	RMsize	RMratio
***Affiliative***	*5*.*333*	*0*.*729*
**Agonistic**	4.714	0.679
**Arousal**	4.526	0.817
**Feeding**	3.167	0.596
***Grooming***	*1*.*500*	*0*.*450*
**Locomotion**	3.000	0.500
***Playing***	*4*.*967*	*0*.*762*
**Resting**	0.000	0.000
***Sex***	*2*.*000*	*0*.*667*
**Unsure/Unknown**	0.000	0.000

A value of 0 for RMsize or RMratio means that no instances of RFM were observed in this category. Categories previously studied are italicized.

**Table 2:** Average RMsize and RMratio scores based on facial signal type.

Facial signal type	RMsize	RMratio
**Ambiguous face**	3.000	0.511
***Bared-teeth face***	*4*.*857*	*0*.*762*
**Lipsmacking face**	2.000	0.625
**Lower lip relaxer face**	3.000	0.500
**Pant hoot face**	4.300	0.799
***Relaxed open-mouth face***	*5*.*217*	*0*.*802*
**Pout face**	0.000	0.000
**Raspberry face**	0.500	0.100
**Scream face**	5.333	0.657

A value of 0 for RMsize or RMratio means that no instances of RFM were observed. Categories previously studied are italicized.

**Table 3:** Average RMsize and RMratio scores based on the number of AUs present in a given facial signal.

Number of aUs	RMsize	RMratio
**1**	0.000	0.000
**2**	1.500	0.750
**3**	0.750	0.250
**4**	2.600	0.650
**5**	4.000	0.800
**6**	4.391	0.732
**7**	5.421	0.774
**8**	5.071	0.634
**9+**	7.000	0.778

## Discussion

In this study, we evaluate whether different types of facial signals and generalized behavioral contexts, beyond those that have been previously studied, exhibit evidence for RFM in chimpanzees (***hypotheses 1 and 2***). We also examined whether RFM is more likely to occur with facial signals that involve fewer facial muscle movements (***hypothesis 3***). Due to the varying precision of RFM, ranging from type matching to exact matching, we used two recently established measures to quantify the extent of RFM: RMsize and RMratio (Martvel et al. 2024). By assessing these hypotheses, we gain further insight into the social function of FM, particularly regarding how it enables chimpanzees to connect both physically and possibly emotionally with other members of their species.

We found no evidence to support ***hypothesis 1***. There were no significant differences in average scores for RMsize and RMratio between previously studied contexts that have confirmed instances of RFM (affiliation, play, grooming, and sex) and new contexts included in our current study. While the reported RMsize and RMratio for play were high (at 4.967 and 0.762, respectively), other contexts (such as agonism and arousal) were also associated with higher average RMsize and RMratio values. This suggests that RFM may occur in various contexts in chimpanzees, serving as a more generalized mechanism for reducing uncertainty during social interactions (Kret and Akyüz 2022). Moreover, we found no evidence to support ***hypothesis 2***. There were no significant differences in the average scores for RMsize and RMratio between previously studied facial signal types that have confirmed instances of RFM (bared-teeth face and the relaxed open-mouth face) and new signal types included in our study. Although the relaxed open-mouth (i.e., play) face had the highest reported RMsize (5.217) and RMratio (0.802), these scores were not significantly different from those of other facial signal types, such as the pant hoot and scream face. These results indicate that RFM may occur in various contexts and with different types of facial signals. These findings are consistent with existing human literature, which indicates that a variety of facial signals are subject to RFM (Dimberg 1982; Moody et al. 2007; Kret and Akyüz 2022). If facial signals serve as predictors of future behavior (Waller et al. 2017), and RFM functions to reduce uncertainty (through behavioral matching; Kret and Akyüz 2022), it stands to reason that even those signals not typically associated with prosocial behaviors in chimpanzees are impacted by RFM. The ability to connect physically and potentially emotionally through these facial signals enhances understanding of how chimpanzees might respond in situations that present higher social risks and potential fitness costs (Nesse 1990), particularly in contexts involving anger and fear. Differences in the prevalence of RFM may be contingent not on the features of the communicative interaction but rather on the social bond quality between two chimpanzees. Previous studies have identified links between pair bond strength and the prevalence of FM across various species (Palagi et al. 2015; Florkiewicz et al. 2018; Palagi et al. 2019a, 2019b; Taylor et al. 2019). This pattern likely includes other primates, such as chimpanzees, that establish and maintain numerous affiliative bonds within a given social group (Escribano et al. 2022).

We discovered that there was no relationship between the complexity of facial signals (i.e., the number of discrete facial muscle movements, or AUs, in a given signal) and RMratio (***hypothesis 3***). Interestingly, RMsize increased as the number of distinct facial muscle movements, or action units (AUs), within a given signal seemed to increase. Chimpanzees have a large and complex facial signaling repertoire compared to hylobatids, likely used to navigate various types of social interactions (Florkiewicz et al. 2024). RFM may be even more important to ensure that the subtleties differentiating these signals are captured to minimize communicative errors; however, this concept should be tested empirically in the future. There are many possible socio-cognitive explanations for how chimpanzees achieve RFM when producing complex facial signals that could be tested in future studies. It is possible that chimpanzees exhibit greater cognitive flexibility than hylobatids (Liebal 2016), allowing them to identify and perform facial muscle movements more efficiently. Furthermore, the mechanism behind these movements may be fine-tuned through social learning, particularly if RFM is practiced early in development. Chimpanzees may have more opportunities than hylobatids to practice RFM due to their differences in social organization (Ryne 1996; Campbell et al. 2010; Florkiewicz et al. 2024). The notion that chimpanzees have facial musculature that better supports facial movements may not be relevant, especially considering that both humans and hylobatids possess a higher proportion of slow-twitch facial muscles (Burrows et al. 2014, 2016). This shared characteristic enables both species to maintain better fine-tuned motor control of their facial signals. This indicates that a primates' ability to perform RFM could be shaped by its socio-ecology rather than anatomical abilities/limitations.

One of the major limitations of our study is that we do not evaluate the impact of social bond strength on the prevalence of RFM among chimpanzees. Social bonding data is available only for 2017 data, while the data from 2018 and 2019 lacks this information. Out of 1,090 observations of facial signaling in chimpanzees, 261 observations were relevant to the analysis of social bond strength; however, 47 of these observations could not be included in the RFM analysis, as they consisted solely of instances where the initiating chimpanzee produced only one facial signal without any corresponding response from the recipient. Future studies should investigate if RFM in chimpanzees is influenced by bond strength, similar to many other social mammals that often engage in affiliative interactions (Palagi et al. 2015; Florkiewicz et al. 2018; Palagi et al. 2019a, 2019b; Taylor et al. 2019). Future studies should also examine the prevalence of DFM across various facial signal types and behavioral contexts, and whether this prevalence is mediated by the strength of social bonds, since our study focused exclusively on instances of RFM.

At the very least, our results highlight the necessity for further research on FM (both rapid and delayed) across different species, types of facial signals, and social contexts. Most research on FM has primarily focused on a restricted set of facial signal types and social contexts. However, it is plausible that FM is a more widespread phenomenon than previously thought, given the results of our current study. The most promising areas to explore FM outside of play are among species that already have documented instances of this behavior (for a review, see Davila-Ross and Palagi 2022; Cordoni et al. 2024; Maglieri et al. 2024). Even if FM does not occur in other signal types and contexts within a particular species, this absence may be due to unique socio-ecological factors. Therefore, comparative research is needed to better understand FM across different species. Our current study focused on RFM because it has been extensively documented in great apes like chimpanzees. However, further studies on delayed FM (DFM) are also necessary (Palagi et al. 2019a, 2019b; Davila-Ross and Palagi 2022; Martvel et al. 2024), as it is likely that this phenomenon occurs across various species, facial signal types, and contexts. Finally, our current study focused specifically on a single troop of chimpanzees. We believe it's important to broaden this research to include other troops of chimpanzees. Doing so will enable researchers to better assess the impact of social learning on FM and its different types.

## Authors’ contributions

Both authors, B.N.F. and T.L., contributed equally to the conceptualization, investigation, and visualization of this project. Both authors collaborated on writing, editing, and reviewing the manuscript. B.N.F. developed the methodology for the study and was responsible for data curation, while T.L. handled the software and data analysis.

## Data Availability

Our raw data, along with our Python and R code, can be found in the electronic supplement.
